# Regenerative medicine for skeletal muscle loss: a review of current tissue engineering approaches

**DOI:** 10.1007/s10856-020-06476-5

**Published:** 2021-01-21

**Authors:** Benjamin Langridge, Michelle Griffin, Peter E. Butler

**Affiliations:** 1 Department of Plastic & Reconstructive Surgery, Royal Free Hospital, London, UK; 2 Charles Wolfson Center for Reconstructive Surgery, Royal Free Hospital, London, UK; 3 Division of Surgery & Interventional Science, University College London, London, UK

## Abstract

Skeletal muscle is capable of regeneration following minor damage, more significant volumetric muscle loss (VML) however results in permanent functional impairment. Current multimodal treatment methodologies yield variable functional recovery, with reconstructive surgical approaches restricted by limited donor tissue and significant donor morbidity. Tissue-engineered skeletal muscle constructs promise the potential to revolutionise the treatment of VML through the regeneration of functional skeletal muscle. Herein, we review the current status of tissue engineering approaches to VML; firstly the design of biocompatible tissue scaffolds, including recent developments with electroconductive materials. Secondly, we review the progenitor cell populations used to seed scaffolds and their relative merits. Thirdly we review in vitro methods of scaffold functional maturation including the use of three-dimensional bioprinting and bioreactors. Finally, we discuss the technical, regulatory and ethical barriers to clinical translation of this technology. Despite significant advances in areas, such as electroactive scaffolds and three-dimensional bioprinting, along with several promising in vivo studies, there remain multiple technical hurdles before translation into clinically impactful therapies can be achieved. Novel strategies for graft vascularisation, and in vitro functional maturation will be of particular importance in order to develop tissue-engineered constructs capable of significant clinical impact.

## Introduction

Skeletal muscle has limited ability to regenerate after injury, with volumetric muscle loss (VML) resulting in tissue fibrosis, disfigurement and chronic disability [1, 2]. VML can occur after a wide range of insults including traumatic injury, ischaemia and tumour resection, however its incidence is not well documented [2, 3]. Civilian trauma data does not directly track VML injury rates, however, of the 150,000 open fractures that occur in the United States each year, the majority of these involve soft tissue loss and ~58% of severe open tibial fractures occur with significant muscle damage [4, 5]. Military data from recent conflicts have highlighted the long-term morbidity resulting from such injuries, with VML accounting for 65% of disability following severe open tibial fractures, and a lifetime disability cost of between $340,000 and $440,000 per patient, independent of medical costs [1].

The current standard of care for VML includes free-flap transfer with muscle tissue to cover soft tissue deficits, with bracing and extensive rehabilitative physiotherapy [2]. Functional muscle transfer including vascular and neural innervation is rare due to the specialist expertise required. Despite these interventions, recovery from such injuries is invariably poor, with significant long-term disfigurement and disability being common [1, 6–8]. Furthermore, tissue transfer techniques have a host of disadvantages including donor site morbidity, limited availability of donor tissue, and the requirement for long-term immunosuppression in allo-transplantation [2].

The burden of morbidity due to recent military conflicts has highlighted the need for novel strategies in the treatment of VML. Tissue engineering approaches have the potential to revolutionise the field through the production of biomimetic skeletal muscle and the manipulation of endogenous regeneration mechanisms. Herein we review the current status of tissue engineered skeletal muscle and its translation into clinically useful therapeutic strategies for VML.

## Pathophysiology of volumetric muscle loss

**Fig. 1:**
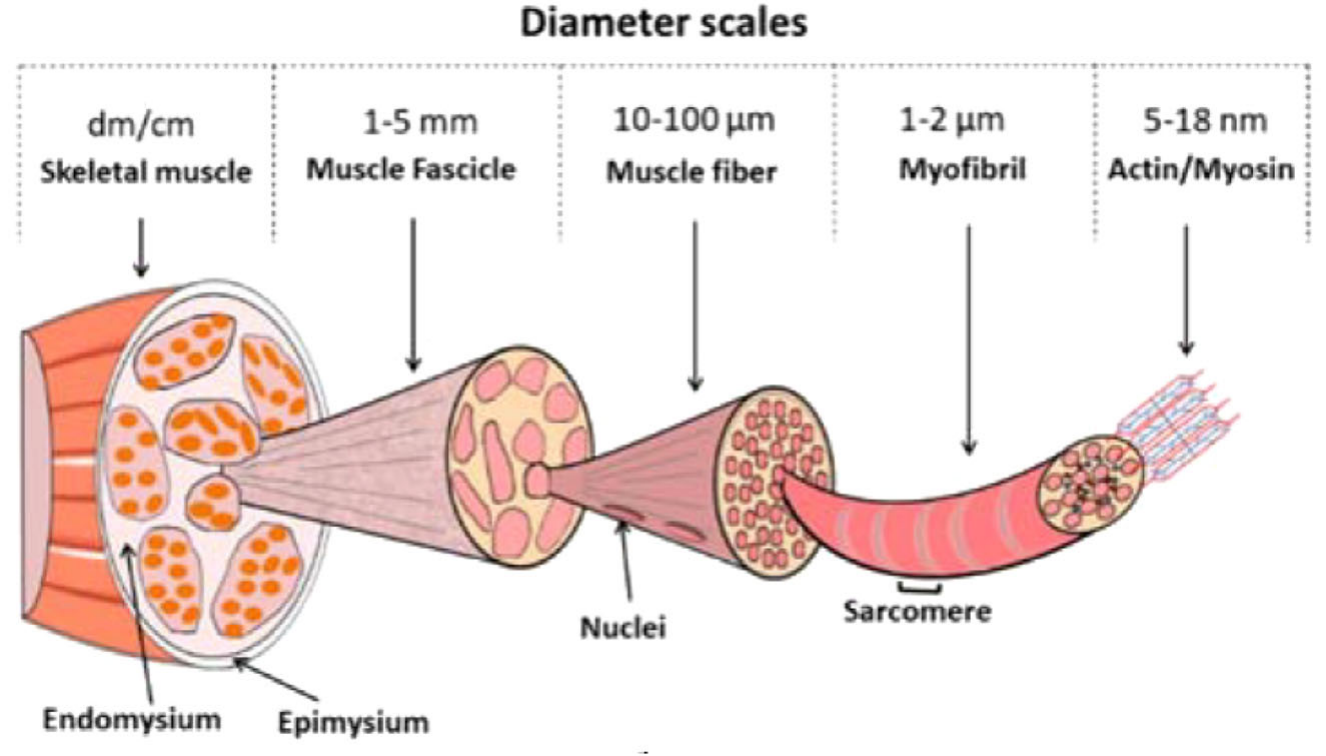
Structure of skeletal muscle (Modified from Beldjilali-Labro et al. [34] under Creative Commons License)

Skeletal muscle is a highly anisotropic structure with the extracellular matrix (ECM) being critical for the both its development in utero and its physiological function in vivo (Fig. 1).

Skeletal muscle commonly incurs injury from everyday activity, yet has a remarkable ability to regenerate through a cycle of inflammation, followed by the myoblastic differentiation of resident satellite cells [9, 10]. In VML however, frank loss of muscle tissue disrupts the tissue architecture beyond that which endogenous mechanisms are capable of repairing, instead resulting in a pathological response characterised by pronounced inflammation, tissue fibrosis, and a chronic loss of muscle and limb function [11, 12].

Clinical evidence from patients with VML is predominantly limited to case reports, providing a poor insight into the underlying pathological mechanisms. These clinical cases demonstrate that after limb salvage, fracture repair and extensive physical rehabilitation, a persistent loss of muscle bulk combined with tissue fibrosis and tethering results in functional deficits due to the reduced torque production, along with the restricted active and passive range of movement [8, 11]. Analysis of the relationship between strength deficits and muscle loss has suggested a non-linear relationship in VML, however, the precise contribution of other pathological mechanisms, such as tissue fibrosis, are yet to be understood [12].

**Fig. 2:**
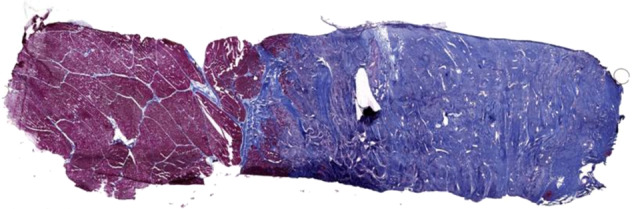
Histological sample from a porcine model of VML by Greising et al. [13]. VML injury was created through surgical excision of porcine peroneus tertius muscle, with histological samples taken at 12 weeks after injury. Significant fibrosis infiltrating into native muscle is seen. Masson’s Trichrome stained sample (Connective tissue is blue; nuclei are purple; skeletal muscle fibres are red). Reproduced under Creative Commons License

In vivo studies have demonstrated that this failure of muscle regeneration occurs in a pathological environment of persistent inflammation and extensive tissue fibrosis [13]. Tissue fibrosis continues to take place for weeks after the initial injury, with invasion into the adjacent uninjured muscle, tethering to skin and fascia, and persistent functional deficit (Fig. 2) [13]. However, comparatively little is known about the molecular mechanisms involved in VML. Studies have demonstrated the persistent activation of acute inflammatory pathways, such as the complement system, along with Wnt and transforming growth factors (TGF-Beta) signalling, with a subsequent attenuation in satellite cell proliferation, and the increased deposition of collagen by fibroblasts [14–17].

Whilst it is apparent that central to the pathogenesis of VML is this persistent imbalance between pro-fibrotic and regenerative pathways, the molecular and cellular orchestration of this process remains poorly understood. In vivo studies have provided some answers, however direct evidence from human studies remains limited. For tissue-engineered therapies to be successful, a clearer understanding of the mechanisms driving this pro-fibrotic microenvironment will be necessary.

## Scaffold use for skeletal muscle engineering

The loss of the highly organised skeletal muscle ECM following VML makes the retention of cell-based therapies and the development of functional tissue a challenge. Tissue scaffolds assist cell delivery by replicating the native ECM, then support the development of muscle tissue by providing structural support, cell adhesion molecules and the release of growth factors [18]. The use of tissue scaffolds has been demonstrated to support the survival and maturation of myoblasts both in vitro and in vivo [19].

The ideal scaffold recapitulates the native skeletal muscle microenvironment; anisotropic three-dimensional (3D) scaffolds provide a biomimetic microarchitecture, topographical cues, along with cellular adhesion molecules that are necessary for muscle progenitor cells to differentiate and organise into functional muscle tissue [20]. Scaffold biocompatability is a key requirement to avoid foreign body type reactions, with tissue fibrosis, scaffold encapsulation and resultant graft failure. The mechanisms of biocompatability are incompletely understood, however, experience with implanted medical devices and organ transplantation has demonstrated the importance of autologous organic materials or inert inorganic materials [21, 22]. Replicating the mechanical properties of the native ECM is also important, several studies have demonstrated that optimal myogenesis occurs on scaffolds with muscle like stiffness [23].

A diverse array of methods have been used in scaffold design, herein we group scaffolds by the method of production and describe their relative merits, with recent examples of their use in skeletal muscle scaffolds described in Table 1.

**Table 1:** Key scaffold types and examples of their use in skeletal muscle constructs

Author	Scaffold material	Cell type	In vivo/in vitro	Outcomes
Decellularised tissue				
Quarta et al. [110]	Murine tibialis anterior (TA) muscle	Satellite cells, endothelial cells, hematopoietic cells, fibroblasts and fibro-adipogenic progenitors	In vivo	Non-myoblastic cell types support satellite cell survival. Perfusion of tissue constructs in vitro improves satellite cell survival. Tissue constructs combined with exercise increased in vivo muscle mass, force generation and murine gait.
Alvarez Fallas et al. [25]	Murine diaphragm muscle	N/A	In vivo	Decellularised scaffold promoted greater neovascularisation and provoked a more limited foreign body reaction than unmodified synthetic PTFE scaffold.
Shapiro et al. [24]	Rabbit skeletal muscle conjugated with IGF-1	Murine C2C12	In vitro	IGF-1 increased C2C12 infiltration into decellularized scaffolds and supported C2C12 proliferation on scaffold.
Hydrogel				
Kim et al. [117]	Fibrinogen, gelatin, hyaluronic acid and glycerol cellularised hydrogel. Glycerol hydrogel sacrificial microchannels. Poly(ε-caprolactone) (PCL) supporting pillar	Human muscle progenitor cell isolate	In vivo	Tissue construct treated VML rats recovered to >80% muscle force generation by week 8. 3D printed constructs regenerated more muscle mass, greater force generation and superior muscle histology than non-printed constructs
Prüller et al. [80]	Collagen I, Fibrin and PEG-Fibrinogen hydrogels	Murine C2C12 myoblasts Immortalised human myoblasts Murine satellite cells	In vitro	Satellite cells transplanted with their cellular niche had superior proliferation and terminal differentiation than those expanded in vitro. Myogenic differentiation occurred on all scaffolds but cell behaviour differed by scaffold material
Han et al. [81]	Poly(ethylene glycol) hydrogel embedded with Wnt7a	Murine satellite cells in vivo Murine C2C12 myoblasts in vitro	In vivo	Wnt7a promotes satellite cell migration into the scaffold and muscle fibre hypertrophy
Nanofiber				
Bloise et al. [82]	Electrospun poly(butylene 1,4-cyclohexandicarboxylate-co-triethylene cyclohexanedicarboxylate) (P(BCE-co-TECE))	Murine C2C12 myoblasts	In vivo	Addition of TECE improved C2C12 proliferation in vitro. The majority of cells populating the scaffold in vivo were inflammatory cell types.
Ribeiro et al. [83]	Electrospun poly(vinylidene fluoride) (PVDF)	Murine C2C12 myoblasts	In vitro	PVDF nanofibers demonstrated piezoelectric properties that promoted fusion and maturation of myoblasts and varied with polarity
Zahari et al. [130]	Electrospun poly(methyl methacrylate), coated with collagen or laminin	Mixed human fibroblasts and myoblasts	In vitro	Genipin increases nanofiber adsorption of collagen and laminin. Laminin coated scaffolds preferentially support myoblast proliferation and migration.
Electroconductive				
Du et al. [43]	Poly (citric acid-octanediol-polyethylene glycol)(PCE)-graphene (PCEG) nanocomposite	Murine C2C12 myoblasts	In vivo	Addition of reduced graphene oxide (RGO) improved scaffold mechanical properties and electrical conductivity. Addition of RGO increased scaffold myofiber and capillary density in vivo
Zhang et al. [84]	SF/PASA: Silk fibroin with poly(aniline‐co‐N‐(4‐sulfophenyl) aniline)	Murine L929 fibroblast and C2C12 myoblasts	In vitro	Characterisation of scaffold electroconductivity and biodegradability. Increasing PASA content enhanced myogenic differentiation of C2C12 myoblasts
Ostrovidov et al. [69]	Gelatin-polyaniline (PANI) electrospun nanofibers	Murine C2C12 myoblastis	In vitro	The addition of PANI increased nanofiber electroconductivity by 10^4^ S/cm. Electrical stimulation of conductive nanofibers enhanced myoblast functional maturation

### Decellularised scaffolds

Decellularised scaffolds are derived from xenogeneic, allogenic or autogenic skeletal muscle tissue [24–26]. Once deceullularisation has removed cellular material, the remaining ECM retains the native 3D microstructure, molecular composition and growth factors that support skeletal muscle regeneration [25]. Decellularization protocols vary between studies but commonly include the use of detergents and enzymes, such as DNase and Trypsin; the effectiveness of the decellularization protocol is central to minimising scaffold immunogenicity [27–31].

Decellularised scaffolds have the advantage of a ready-made, tissue-specific ECM with the appropriate microarchitecture and molecular composition. Their ability to natively support myogenesis and angiogenesis is a significant advantage over synthetic scaffolds which require extensive development to gain similar characteristics [25]. However, decellularised scaffolds are dependent on the availability of appropriate donor tissues, carry a risk of contamination with pathogenic organisms, cause donor morbidity, and ideally need to be autologous to minimise the risk of immunogenicity [22, 32, 33].

### Hydrogels

Hydrogels are a family of hydrophilic polymers with a high-water content consisting of either natural or synthetic materials. Natural hydrogels consist of materials, such as collagen, fibrin, chitosan and hyaluronic acid; they are biodegradable, but have limited mechanical strength and can provoke an immune response in vivo [34, 35]. Synthetic hydrogels, such as polyethylene glycol have superior mechanical properties that can be tailored more readily however, as they inherently lack biological molecules, they require modification to support cell adhesion, differentiation and viability.

The mechanical properties of hydrogels have been well characterised and they can be mixed to produce a composite hydrogel with superior properties for tissue engineering. Collagen type 1, for example, is ubiquitous in the ECM and has good mechanical properties for skeletal muscle tissue engineering, such as significant mechanical stretch before failure, and its interconnected fibres and small internal pore structure limit cellular migration whilst permitting diffusion of oxygen and nutrients [36–38].

**Fig. 3:**
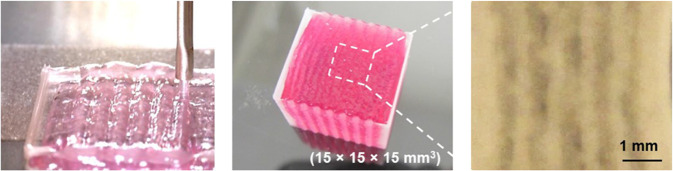
3D bioprinting of a skeletal muscle construct with sacrificial gelatin hydrogel components to generate microchannels within the construct. These microchannels facilitate the diffusion of oxygen and nutrients to cells at the centre of the construct. (Modified from Kim et al. 2018 [117] under Creative Commons License)

Hydrogels are also highly suitable for the entrapment of cells and biomolecules, such as growth factors, that promote cellular survival, myogenic differentiation and angiogenesis within the hydrogel. Effective engineering of the hydrogel microenvironment can create an artificial niche ideal for skeletal muscle regeneration; techniques, such as photolithography can pattern hydrogels to create spatial variations within the scaffold capable of guiding cellular behaviour and the layered deposition of hydrogels with differing mechanical properties can be used to control tissue microarchitecture (Fig. 3) [39]. Control of the temporal dynamics of entrapped biomolecules within a hydrogel is useful for promoting sequential processes, such as cellular differentiation but is technically challenging; multiple approaches have been described, such as the use of double-layered nanospheres capable of sequentially releasing biomolecules in a programmed order [40].

### Nanofibers

Nanofibrous scaffolds are defined as a mesh of nanoscale (0–100 nm) synthetic fibres, these can closely mimic the architecture of the native ECM. Nanofibers can be manufactured via several methods including thermal cycling and phase separation, or electrospinning. Electrospinning is widely used in skeletal muscle tissue engineering given the ability to produce anisotropic, geometrically aligned nanofibers capable of guiding the formation of aligned myofibers [41].

Similar to hydrogels, nanofibrous scaffolds can be utilising both natural materials, such as Collagen, or artificial polymers, such as Polycaprolactone (PCL), these can additionally be combined with hydrogels to leverage both materials’ characteristics in a core-shell arrangement [42]. The combination of these two material types provides the topographical cues of nanofibers to guide myofiber differentiation and alignment, with the ability of hydrogels to provide a microenvironment conducive to prolonged cellular survival [42].

Nanoscale materials can be utilised to modify the properties of nanofiber scaffolds in unique ways to improve mechanical properties, wettability, cellular adhesion, cellular differentiation and electroconductivity [43–45]. Electroconductive scaffolds are presently of particular interest in skeletal muscle tissue engineering.

### Electroconductive scaffolds

In vivo, skeletal muscle receives motor neuron innervation which, via the neuromuscular junction, causes cell membrane depolarisation and myofiber contraction. This electrochemical stimulation not only affects mature myofiber function, but is also necessary for normal myoblast differentiation during embryonic development [46]. In vitro, electrical stimulation of skeletal muscle tissue constructs improves myocyte functional maturation and contractility [47–49]. However, not all electrical stimulation is equal in utility, and the development of an optimal electrical stimulation protocol for myofiber development is ongoing [49–54].

Electroactive scaffolds have been developed through the incorporation of carbon nanotubes, graphene, metals and conductive nanopolymers to make novel nanocomposites. Carbon nanotubes are of interest as they are renowned for their remarkable strength, elasticity and electrical conductivity [55–57]. Ramón-Azcón et al. demonstrated the use of dielectrophoresis to produce a hydrogel containing anisotropic carbon nanotubes, thereby making the hydrogel’s mechanical strength, electrical conductivity and anisotropy more suitable for skeletal muscle scaffolds [58]. Despite the interesting properties of carbon nanotubes however, concerns over their potential toxicity need to be resolved before they can be useful in human studies [59].

Like carbon nanotubes, graphene has excellent electrical conductivity and mechanical strength and has been used to make electroactive nanocomposite scaffolds. The incorporation of graphene and its derivatives, such as graphene oxide, with hydrogels to produce electroconductive composite scaffold has been demonstrated to improve myoblast proliferation, differentiation, organisation and functional maturation [43, 60, 61]. Whilst graphene appears not to be cytotoxic in vitro, its non-biodegradable nature presents a possible toxicity risk in vivo, thus further in vivo investigation is required to establish its safety and biocompatibility profile if it is to be deployed in human trials [62].

Nanocomposites incorporating metals, such as gold and silver are also of interest given their electrical conductivity and have been incorporated in nanoparticle and nanofiber forms to enhance hydrogel electrical conductivity [63]. Gold nanoparticles in particular have advantages over carbon nanotubes and graphene given their electroconductivity and mechanical strength, albeit with a well-established safety profile for use in humans [64].

Conductive nanopolymers are a distinct class of highly versatile polymers that can be incorporated into composite hydrogels or electrospun nanofibers and whose electroconductive and biodegradable properties can be tailored; over 25 different conductive polymers have been described [65]. Polyaniline has been combined with polymers, such as PCL and electrospun into nanofibers to create electroconductive, anisotropic scaffolds that enhance myoblast differentiation and functional maturation [66–69]. Similarly, polypyrrole and polythiophene derivative-based nanopolymers have also been used [70, 71]. Conductive nanopolymers have the additional benefit of being biodegradable and, in many cases, being biocompatible [65, 72, 73].

The development of an optimal scaffold capable of being used in the treatment of human VML is ongoing. However, significant advances have been made in refinement of the materials and processes and our understanding of how to combine them to in order to fine tune the properties of a composite scaffold. Scaffold design is only one part of the solution however; seeding a scaffold with myocyte progenitors in vitro and manipulating the scaffold microenvironment to drive its functional maturation are similarly important, these topics are discussed subsequently.

## Progenitor cells populations for skeletal muscle tissue engineering

Regenerating skeletal muscle myocytes can be derived from endogenous myoblastic cell populations, such as satellite cells, or can be derived from tissue scaffolds prepopulated with myoblasts in vitro. Acellular scaffolds have been used however their utility appears limited, in some cases being completely reabsorbed without any appreciable skeletal muscle regeneration [13, 74]. Pre-population of scaffolds with myoblasts significantly enhances myocyte regeneration, this may in part be due to endogenous satellite cell depletion following VML [17, 26, 74, 75].

The ideal cell population for use in skeletal muscle constructs should be from an accessible source, have high proliferative potential in vitro in order to generate a clinically useful volume of muscle, whilst also retaining the ability to terminally differentiate efficiently into mature myofibers. These myoblasts can be autologous, allogenic or xenogenic however, for clinical use autologous cell sources are most useful due to their non-immunogenicity. Adult somatic cells are terminally differentiated and have restricted ability to undergo mitosis, thus limiting their usefulness in tissue engineering as it is difficult to generate a suitably large population in vitro. Progenitor cell groups have increasing ability to expand with increasing stem-ness and thus have been the primary focus of attention [76]. The primary cell populations used in skeletal muscle tissue engineering are summarised in Table 2.

**Table 2:** Progenitor cell populations used in tissue-engineered skeletal muscle constructs

Cell Type	Origin	Advantages	Disadvantages
Satellite cells	Skeletal muscle	Native stem cell for muscle regeneration in vivo Efficient differentiation Widely used in skeletal muscle tissue engineering	Invasive collection method Low yield isolation processes Senescence causes reduced myogenic potential after expansion in culture
Murine C2C12 myoblasts	Immortalised murine myoblast cell line	Rapid proliferation Efficient differentiation Commercially available Widely used in skeletal muscle tissue engineering	Immunogenicity in vivo
iPSCs	All tissues	Flexible choice of donor tissue Unlimited self-renewal	Highly inefficient process of cellular reprogramming Risk of tumour formation
MSCs	Bone marrow Umbilical cord	High proliferative potential Bone MSC collection is high yield Umbilical MSC collection is non-invasive	Lower myogenic differentiation potential than satellite cells Bone MSC collection is painful and invasive Low availability of autogenic umbilical MSCs
Minced muscle grafts	Skeletal muscle	Simple collection method high yield	Invasive collection method mixed cell types

*iPSCs* induced pluripotent stem cells, *MSCs* mesenchymal stem cells

Satellite cells are the native progenitors for skeletal muscle regeneration in vivo and thus are regularly used in skeletal muscle constructs [77]. In vivo, satellite cells respond to injury by upregulating myogenic transcription factors, such as MyoD and Myf5, thereby being induced into myoblasts which can fuse to form new myotubes, or alternatively fuse with existing damaged myofibers [9]. Satellite cells can be expanded up to 50 times in vitro however, they are challenging to isolate from human skeletal muscle and can lose stem cell potency once ex vivo; development of a medium that can help retain these characteristics would improve their utility [76, 78, 79].

Murine C2C12 are immortalised myoblasts derived from murine satellite cells and are widely used in vitro [24, 43, 69, 80–84]. They readily proliferate and differentiate under differing serum conditions and so are a useful tool however, given that they are xenogenic, they are not appropriate for clinical translation. Furthermore, some studies have reported differences in the behaviour of these cells compared to human myoblast populations [85].

Human induced pluripotent stem cells (iPSCs) are capable of unlimited self-renewal and have been used to derive myogenic progenitor cells, these have subsequently been differentiated into contractile myotubes and satellite-like cells [86, 87]. Rao et al. demonstrated that these myotubes can mature in 3D culture, with increasing force production during contraction, and are also able to integrate with existing muscle and vascularise when in vivo [88]. iPSCs may thus be a viable alternative to satellite cells however, protocols for their efficient terminal differentiation and minimising the risk of tumourigenesis require further research [89].

A diverse range of other cell types have also been utilised with variable success including stem cells derived from adipose tissue, bone marrow, umbilical cord mesenchyme, along with induced stem cells from tissues, such as dermal fibroblasts [90, 91]. Despite the plethora of cell types that could be used to derive myogenic cell lines, many are yet to be studied in detail in skeletal muscle tissue engineering.

Whilst murine progenitors have been important in research thus far, clinical translation of tissue-engineered skeletal muscle will require autologous cell populations to eliminate the risk of immunogenicity. Autologous iPSCs are theoretically excellent candidates for clinically deployable tissue constructs but require further investigation if reliable, efficient and safe myogenesis is to be achieved.

## Scaffold maturation in vitro

The development of a skeletal muscle construct capable of regenerating VML is a challenging, multistage process and many studies utilise a period of in vitro tissue culture to promote myoblast proliferation and the functional maturation of their construct. Approaches have included the addition of growth factors, co-culture with supportive cell types, mechanical stretch and electrical stimulation.

Many growth factors have been used to enhance myogenesis including fibroblast growth factor, hepatocyte growth factor, prostaglandin E2 and insulin-like growth factor, whilst TGF-β1 have been demonstrated to promote functional maturation of scaffolds by enhancing myocyte contractility (Table 3) [76, 92–96]. Furthermore, pro-angiogenic factors, such as vascular endothelial growth factor have been used to improve the vascularisation of skeletal muscle constructs [97].

**Table 3:** Growth factors utilised in the development of skeletal muscle tissue constructs

Growth factor	Effect in vitro
IGF-1	Promotes satellite cell proliferation and differentiation, increases construct force production [94]
FGF	Promotes satellite cell proliferation and differentiation [95]
HGF	Released on muscle injury; promotes satellite cell proliferation, inhibits differentiation [95, 131]
PGE2	Promotes myoblast proliferation [96]
TGF- β	Inhibits satellite cell differentiation, promotes fibroblast proliferation, increases construct contractility [93, 132]
VEGF	Increased tissue construct neovascularisation and myofiber regeneration in vivo [97]

*IGF-1* insulin-like growth factor-1, *FGF* fibroblast growth factor, *HGF* hepatocyte growth factor, *TGF- β1* transforming growth factor beta

Co-culture of myoblasts with complimentary cell types has been demonstrated to promote myogenesis. Fibroblasts proliferate and co-localise with regenerating myofibers in vivo and, whilst fibroblasts are known to play an important role in ECM remodelling, Mackey et al. demonstrated through in vitro co-culture that human fibroblasts additionally promote myoblast differentiation and maturation in a contact dependent manner [98]. Differentiating neural cells have also been demonstrated to support myogenesis when co-cultured with myoblasts [99, 100]. Whilst the addition of cells, such as fibroblasts and neural cells may aid functionalisation of skeletal muscle constructs in vitro, it remains to be seen whether they are required for constructs used in vivo where endogenous populations of these cells are present.

As with electrical stimulation, developing muscle undergoes mechanical stretch in vivo; Vandenburg et al. demonstrated that mechanical tension in vitro promoted myofiber alignment along the axis of tension and stimulates contractile protein accumulation [101]. As with electrical stimulation, a multitude of stretch protocols have been utilised in the literature, often requiring custom-designed equipment to effectively apply them to tissue constructs, but the optimal protocol for preimplantation maturation remains to be established (Table 4) [51, 102–108].

**Table 4:** Mechanical and electrical stimulation protocols used for skeletal muscle construct maturation

Author	Cell type	Scaffold material	Stimulation protocol	Outcomes
Mechanical stimulation				
Aguilar-Agon et al. [104]	C2C12 Myoblasts	Collagen hydrogel	Progressive load to 15% strain over 1 h, 2 h isometric tension.	Myotube diameter and maximal contractile force increased at 45 h post loading.
Heher et al. 2015 [105]	C2C12 Myoblasts	Fibrin hydrogel	10% static strain for 6 h, 3% static strain for 18 h rest period. 6 days training	Strain-induced cell and actin alignment. Increased myotube diameter, length and differentiation.
Candiani et al. 2010 [107]	C2C12 Myoblasts	Electrospun DegraPol®	3 days unidirectional strain (3.3%) followed by 10 days cyclic strain (0.5 Hz, 3.4% strain, 28 min rest period)	Enhanced myosin heavy chain expression in myoblasts subjected to dynamic strain protocol vs. static strain alone
Matsumoto et al. [108]	C2C12 Myoblasts	Fibrin hydrogel	Static strain (0–200%)	Myofibers aligned along the axis of strain. Myoblast proliferation increased with increasing strain
Electrical stimulation				
Patel et al. [52]	C2C12 Myoblasts	Collagen I or Laminin-111	1 V, 2 ms pulses at 2 Hz, 1 h/day for 3 days	Electrical stimulation increased expression of MyoD and myogenin during differentiation.
Khodabukus et al. [53]	Human Myocytes (minced muscle)	Matrigel (BD Biosciences)	70 mA, 2 ms pulses at 1 Hz or 10 Hz. 1 h stimulation, 7 h rest period. 7 days training	Stimulation increased myobundle size, sarcomere proteins and contractility. 10 Hz stimulation resulted in greater hypertrophy vs. 1 Hz.
Ito et al. [49]	C2C12 Myoblasts	Matrigel and collagen	0.3 V/mm, 4 ms pulse width at 1 Hz. 10 days training	4.5-fold increase in force generation at day 14 compared to constructs without electrical stimulation
Donnelly et al. [54]	C2C12 Myoblasts	Fibrin hydrogel	1.25–5 V/mm, 4 pulses of 0.1 ms, 3.6 s recovery, 7 days training	Greatest force production at 2.5 v/mm
Electromechanical stimulation				
Kim et al. [106]	C2C12 Myoblasts	Fibrinogen and matrigel	2.3% strain over 4.3 s. 2.5 v/mm, 1 ms pulses, 0.1–0.5 Hz. In-phase vs. out-of-phase electromechanical stimulation. 3 min or 20 min training.	Out-of-phase electromechanical stimulation resulted in greater improvement in construct force generation than either in-phase, or separate electrical and mechanical stimulation.
Liao et al. [51]	C2C12 Myoblasts	Electrospun diisocyanate-based polyurethane fibres	5% or 10% cyclic strain at 1 Hz for 1 h, 5 h rest period. Electrical stimulation 20 V, 10 ms pulse width. Between days 2 and 14 post differentiation.	Increased MHC and percentage of striated myotubes under electromechanical stimulation. Electrical stimulation at early timepoints was detrimental to myotube development. Cyclic strain alone promoted proliferation over differentiation.

**Fig. 4:**
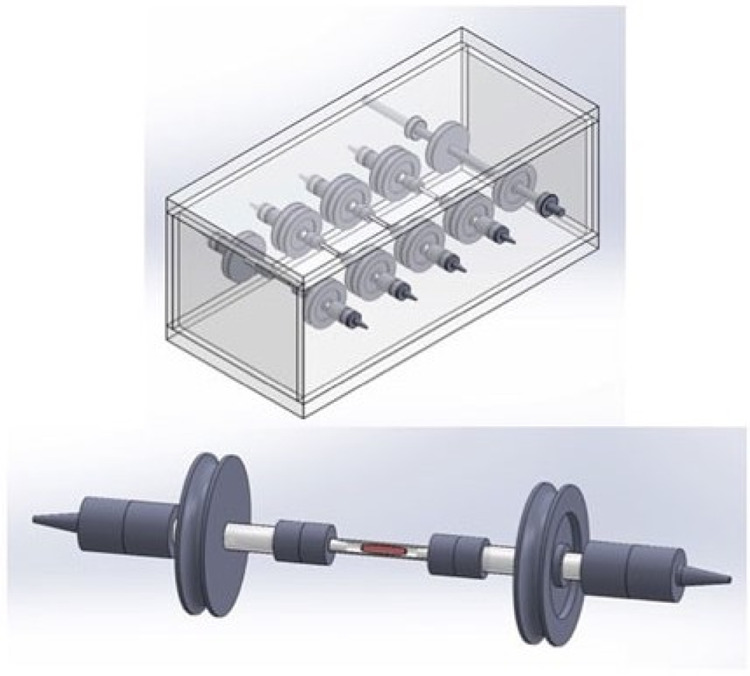
The use of bioreactors to perfuse skeletal muscle tissue constructs improves cellular survival. This bioreactor model permits parallel incubation of multiple tissue constructs (Modified from Quarta et al. [110] under Creative Commons License)

The variety of factors that can influence skeletal muscle construct maturation in vitro, and the need to support metabolically active tissue during this incubation period has led to the development of bioreactors designed to maintain construct homoeostasis and monitor its functional maturation (Fig. 4) [109, 110]. Current bioreactors are rudimentary, however, in order to reach the goal of translating tissue engineering approaches to clinical practice, bioreactors that are reliable, scalable, sterile, and are capable of both monitoring and controlling the tissue construct microenvironment in real time are needed [111]. Some studies have demonstrated bioreactors capable of supporting tissue constructs and monitoring them in real time, however further development is required to ensure adequate control of the metabolic microenvironment [112].

### Three-dimensional bioprinting

The successful translation of skeletal muscle constructs into clinical practice requires the macro-architecture of the construct to be designed around the tissue deficit of the patient. The combination of tissue engineering, imaging modalities and 3D printing technology provides the potential to personalise the macro-architecture of a tissue construct to the clinical need of a patient.

3D printing is a collection of approaches also known as ‘additive manufacturing’ and utilises computer aided design software to design and then print structures through the sequential deposition of layers of material. The ability to 3D print biologically compatible ‘inks’ containing scaffolds, cells and other biomolecules has led to the concept of 3D bioprinting. 3D bioprinting has clear advantages in the manufacturing of personalised tissue constructs however it brings its own technical challenges. Extrusion based printers are the most commonly used, but extrusion exerts significant sheer forces which can damage cells contained in bioinks, and can compress scaffolds, reducing porosity and modifying mechanical properties. The broad range of 3D bioprinting methods, and the methods of translating patient imaging, such as computed tomography into computer models for printing, are outside the scope of this paper but are extensively covered elsewhere [113].

### The musculotendinous junction

**Fig. 5:**
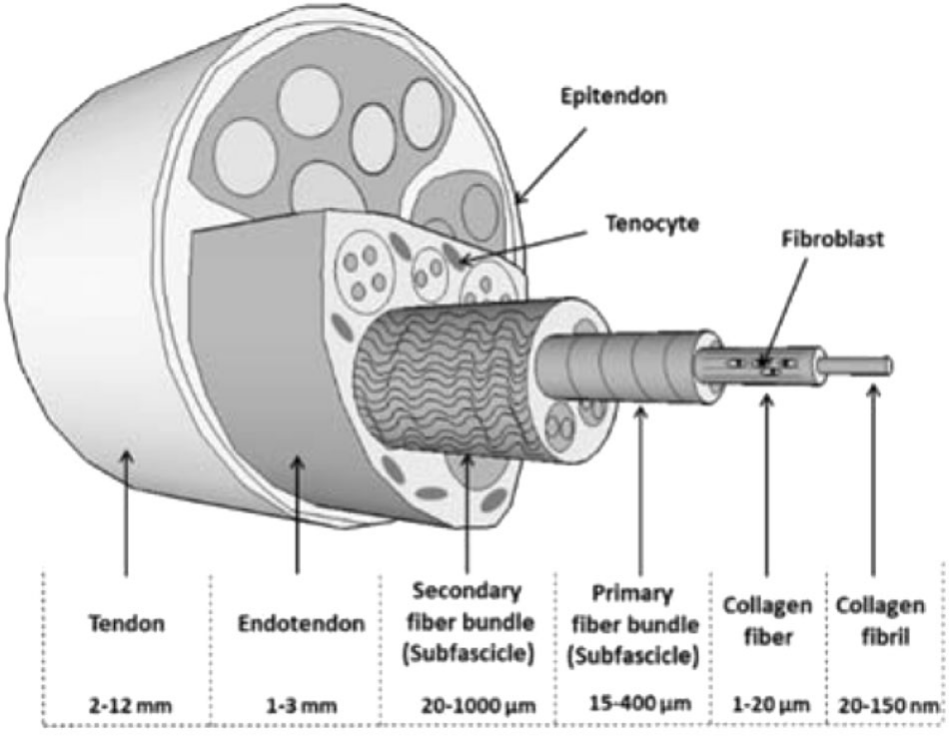
Structure of muscle tendon (Modified from Beldjilali-Labro et al. [34] under Creative Commons License)

The function of skeletal muscle in vivo is dependent upon efficient force transfer from contracting muscle to bone via its tendinous anchorage. Whilst the basic architecture of the musculotendinous junction has been described (Fig. 5), understanding of how this forms in vivo is limited [114]. Attempts to synthesise a skeletal muscle construct with a functioning tendon are infrequent [115, 116]. Whilst a musculotendinous junction is a necessity for the synthesis of a complete muscle body, it may not be required for a clinically useful treatment for VML as skeletal muscle constructs have been demonstrated to integrate with residual native muscle in vivo.

### Progress in vivo

Clinical translation requires in vivo studies to demonstrate a tissue-engineered construct to successfully integrate into a VML site and generate a long-term functional improvement. Most in vivo work thus far been conducted in animal models these have demonstrated the ability of skeletal muscle constructs to integrate with host skeletal muscle and vasculature in vivo, with some studies demonstrating a subsequent improvement in force generation [117–119].

**Fig. 6:**
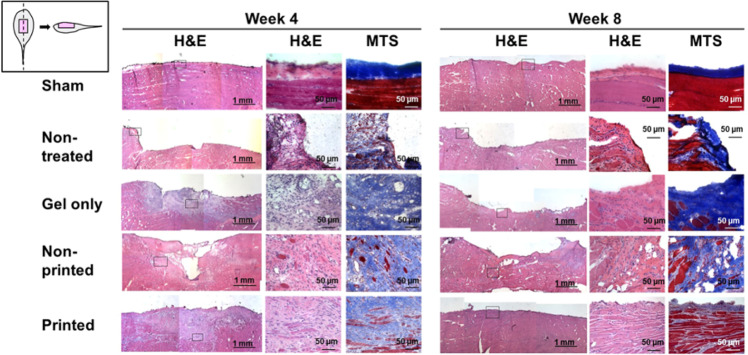
Histological images demonstrating aligned, newly formed myofibers in bioprinted skeletal muscle constructs at 4 and 8 weeks post implantation. (Reproduced from Kim et al. [117] under Creative Commons License)

Quarta et al. demonstrated in a mouse model of VML that skeletal muscle constructs can not only improve active mechanical properties, such as force generation, but also reduce the pathological fibrosis subsequent to VML and revert the pathological length-tension curve to pre-injury characteristics [26]. Separately, Kim et al. demonstrated the use of a bioprinted hydrogel construct consisting of human muscle progenitor cells and sacrificial microchannels to maintain construct viability in vivo prior to neovascularisation, reporting an 82% functional improvement in their rodent VML model (Fig. 6) [117]. Studies, such as these demonstrate the potential for tissue-engineered constructs to generate both anatomical and functional recovery following VML.

## Barriers to clinical translation

### Current state of adoption

Presently, skeletal muscle tissue constructs are predominantly limited to in vitro and in vivo research and attempts to deploy them in human clinical trials have been limited. Acellular scaffolds derived from decellularized animal tissue have been utilised in humans, but their success in regenerating functional muscle tissue has been limited [8, 119, 120]. The development of a clinically useful therapeutic strategy will first require the scaling of several key technical, regulatory and ethical challenges.

### Technical challenges

Scaffold design is a core feature requiring optimisation. Recent developments involving autologous hydrogels, electroactive nanofibers and core-shell composite scaffolds are important, however a scaffold is yet to be demonstrated with the optimum characteristics of biocompatibility, biodegradability and the ability to effectively guide myogenesis in vivo [22, 42].

The optimal autologous stem cell population for use in VML patients is as yet undetermined; whilst much research has utilised muscle-derived stem cell populations, such as satellite cells, the requirement in human patients to generate large volumes of autologous myoblasts to replace VML makes them unsuitable given their limited proliferation in vivo, and challenges, such as senescence after expansion in vitro. iPSCs are an exciting alternative, however, current cellular reprogramming protocols are highly inefficient, and safety concerns regarding in vivo tumorigenesis are yet to be resolved [121].

A further key challenge is that of scalability. The current data from in vivo studies is typically of small tissue constructs ~1 cm^3^ in volume. In order to provide meaningful functional recovery in human VML, significantly larger constructs are required which will need improved solutions for driving construct neovascularisation and innervation. Physiological tissue requires an extensive vasculature network as the maximum diffusion distance of nutrients is ~150–200 μm [122]. Current approaches utilising pro-angiogenic growth factors and sacrificial microchannels are too slow to sustain a large construct [123]. To be effective, large constructs may need to be pre-vascularised prior to implantation. Recent examples from cardiac tissue engineering have used oxygen diffusion modelling to design 3D printed microvasculature; combining approaches, such as this with a period of in vitro maturation may provide a solution [124].

### Regulatory challenges

The transition from tissue constructs used for research, to the industrial manufacture of therapeutic products requires orders of magnitude improvements in speed, efficiency, cost and the standardisation of constructs. The development of closed, automated manufacturing systems is currently limited to rudimentary bioreactors; the further development of such technologies will be central to the production of tissue constructs at an industrial scale, alongside providing the reliability in safety and clinical effectiveness that is required by regulators and clinicians [125].

The precise regulatory status of tissue constructs is unclear within many jurisdictions, however, any product targeting routine use in human patients will need to meet standards, such as Good Manufacturing Practice regulations, as well as being approved by regulatory bodies, such as the European Medicines Agency (EMA) or the Food and Drug administration (FDA). Within the European Union, the EMA regulates tissue-engineered products under advanced-therapy medicinal product regulation, with the FDA classifying them as combination products; to meet the stringent standards of either regulatory body will require thorough testing in clinical trials and may require the development of novel regulatory frameworks [113, 126, 127].

### Physician and patient perception

For tissue-engineered therapies to be adopted in clinical practice, physicians and patients will not only require that the technical and regulatory hurdles are overcome, but also that specific ethical standards are maintained. Stem cell science is a key component of tissue engineering and has a long history of ethical challenges, most notably with the use of human embryonic stem cells; such considerations will be important in the search for a suitable progenitor cell population [128]. Similarly, the use of xenogenic material as in decellularised scaffolds may not be acceptable for some patient groups [128]. Finally, transparency regarding conflicts of interest may be especially pertinent to tissue engineering given the significant overlap in the community of clinicians and scientists that will be necessary for the development, testing and clinical application of these novel therapies [129].

## Conclusions

There is a vast unmet need in the care of patients with VML; current therapeutic approaches provide limited functional and anatomical recovery, and come at a significant cost to healthcare systems. Tissue engineering approaches show significant potential, but many challenges remain to be solved before clinically useful constructs are commercially available; the design of an optimal scaffold, the manipulation of an appropriate progenitor cell population, and the scaling of tissue constructs in both size and speed of production are key technical hurdles. Separately, changes to current regulatory processes may be required if promising therapies are to be translated efficiently. The progress that has been made thus far clearly demonstrates the potential for tissue engineering to revolutionise the treatment of patients with VML; the collaboration of diverse communities of scientists, clinicians and regulators will be needed in order to surmount the challenges that remain.
